# Supplementary material for: Mesophyll porosity is modulated by the presence of functional stomata
Source: Nat Commun. 2019 Jun 27;10:2825. doi: 10.1038/s41467-019-10826-5 (PMC6597550; doi:10.1038/s41467-019-10826-5)
Supplement: Supplementary file 1 — Supplementary Information [file 41467_2019_10826_MOESM1_ESM.pdf]

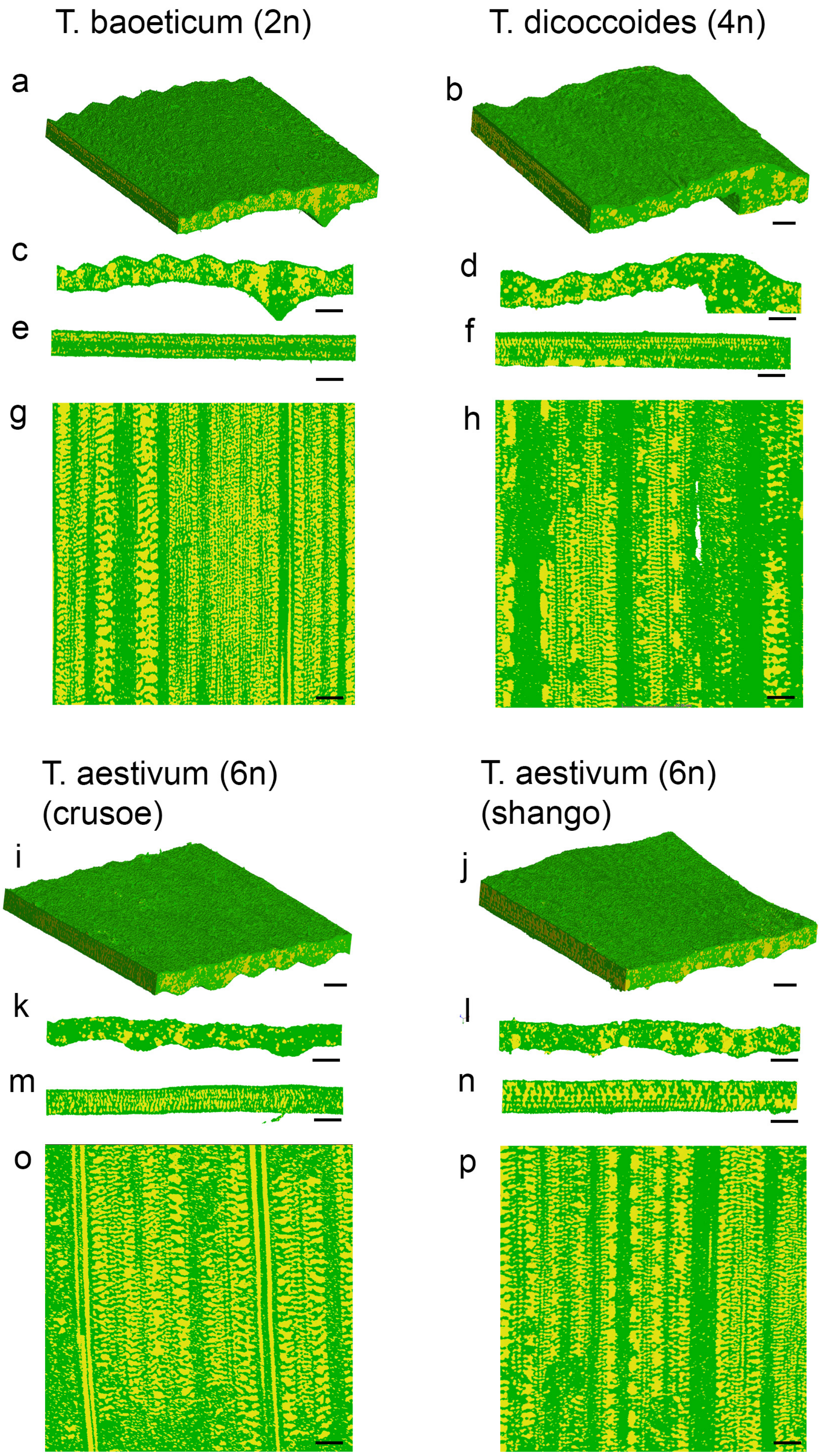

Supplementary Figure 1. MicroCT images of wheat lines. Sample leaf images of *Triticum baoeticum (2n), T. dicoccoides (4n)* and *T. aestivum* cv Crusoe (6n) and cv Shango (6n) in 3D renderings of tissue blocks (**a,b,i,j**), transverse sections (**c,d,k,l**), longitudinal sections (**e,f,m,n**), and paradermal sections (**g,h,o,p**), with solid tissue in green and airspace in yellow. Resolution = 2.75 µm. Scale bars = 1mm.

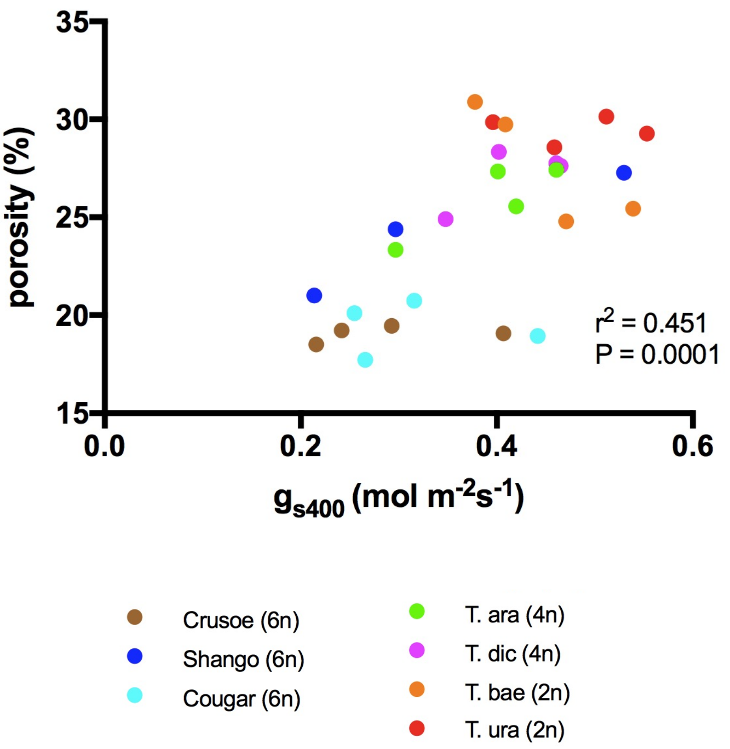

Supplementary Figure 2. Correlation analysis of g_s and porosity for individual leaves from wheat plants of 2n, 4n or 6n ploidy level. Sample identity is indicated in the key. The grouped data (based on ploidy level) are shown in Figure 1I. Pearson correlation coefficient r^2 and P value are shown.

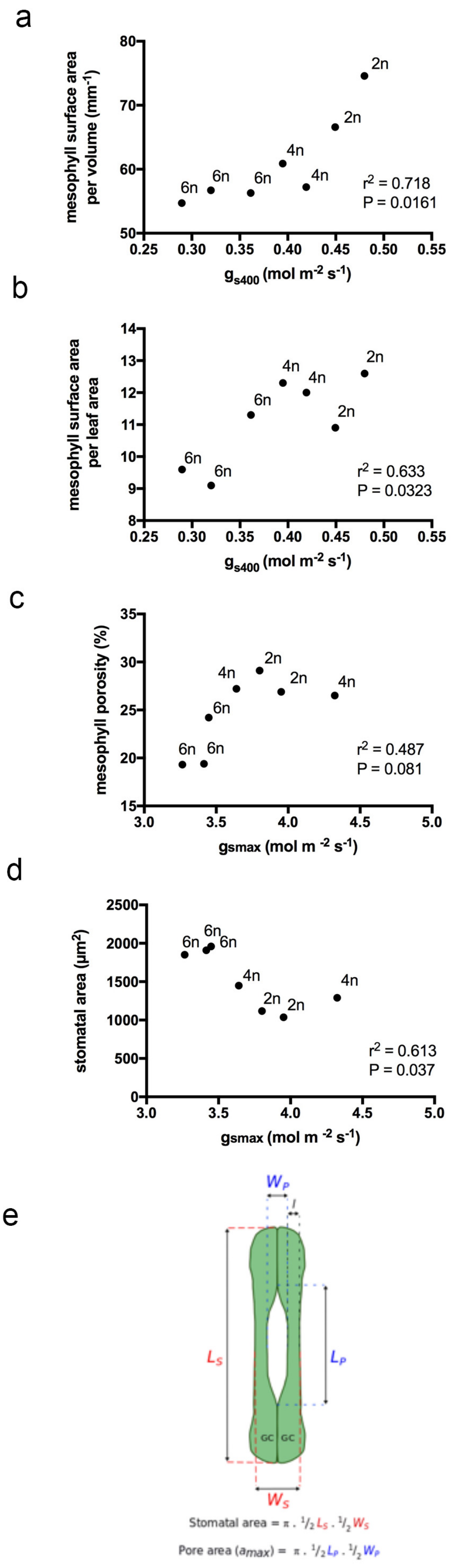

Supplementary Figure 3. Stomatal conductance varies with ploidy level in wheat. (**a**) Mean stomatal conductance, g_s, plotted against exposed internal mesophyll surface area per tissue volume or (**b**) exposed internal mesophyll surface area per one-sided leaf surface area (**c)** mean maximal stomatal conductance to water vapour, g_{smax}, plotted against mean mesophyll porosity or (**d**) mean stomatal area. Data are presented for the wheat lines described in Fig. 1, with ploidy level indicated for each point. Results of correlation analysis (Pearson value r^2) are shown, n=6 for each line. **(e)** Measurements taken for the calculation of g_{smax}.and stomatal dimensions.

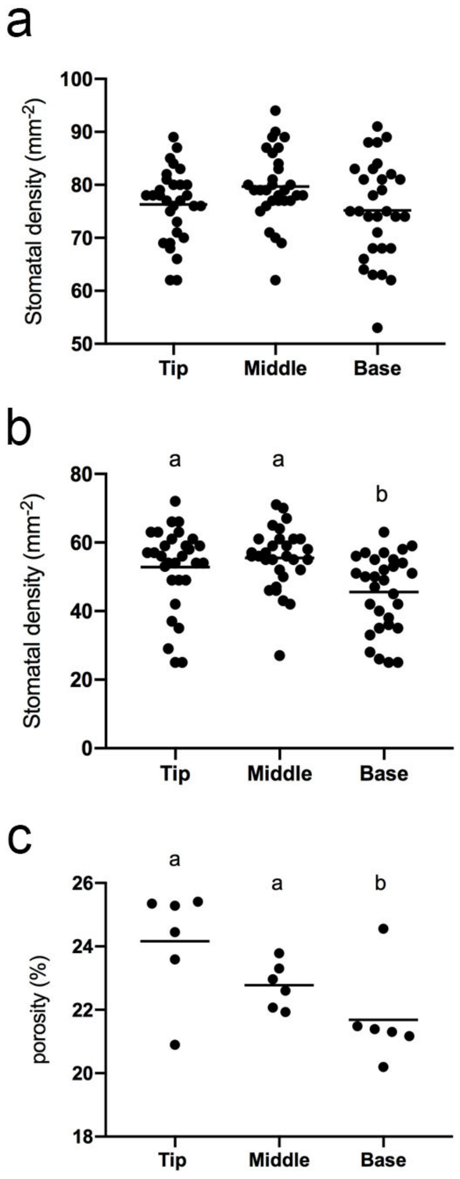

Supplementary Figure 4. Stomatal density and porosity gradients along mature wheat leaves. (a) Stomatal density at the tip, middle and base of mature leaf 5 of *T. aestivum cv* Fielder. **(b)** Stomatal density at the tip, middle and base of mature leaf 5 of TaEPF1 OE plants **(c)** Porosity at the tip, middle and base of mature leaf 5 of TaEPF1 OE plants. Values are shown for individual plants, with horizontal bar indicating the mean value. ANOVA indicated a significant difference in values comparing tip, middle and base regions for both stomatal density ($F_{(,2, 87)}$, P = 0.0019) and porosity ($F_{(2,15)}$, P = 0.024) in the TaEPF1 OE lines. Lines indicated with the same letter cannot be distinguished from each other at the 0.05 confidence limit (Tukey test). No significant variation in stomatal density was detected between the tip, middle and base of the *Fielder* leaves ($F_{(2,87)}$, P = 0.061).

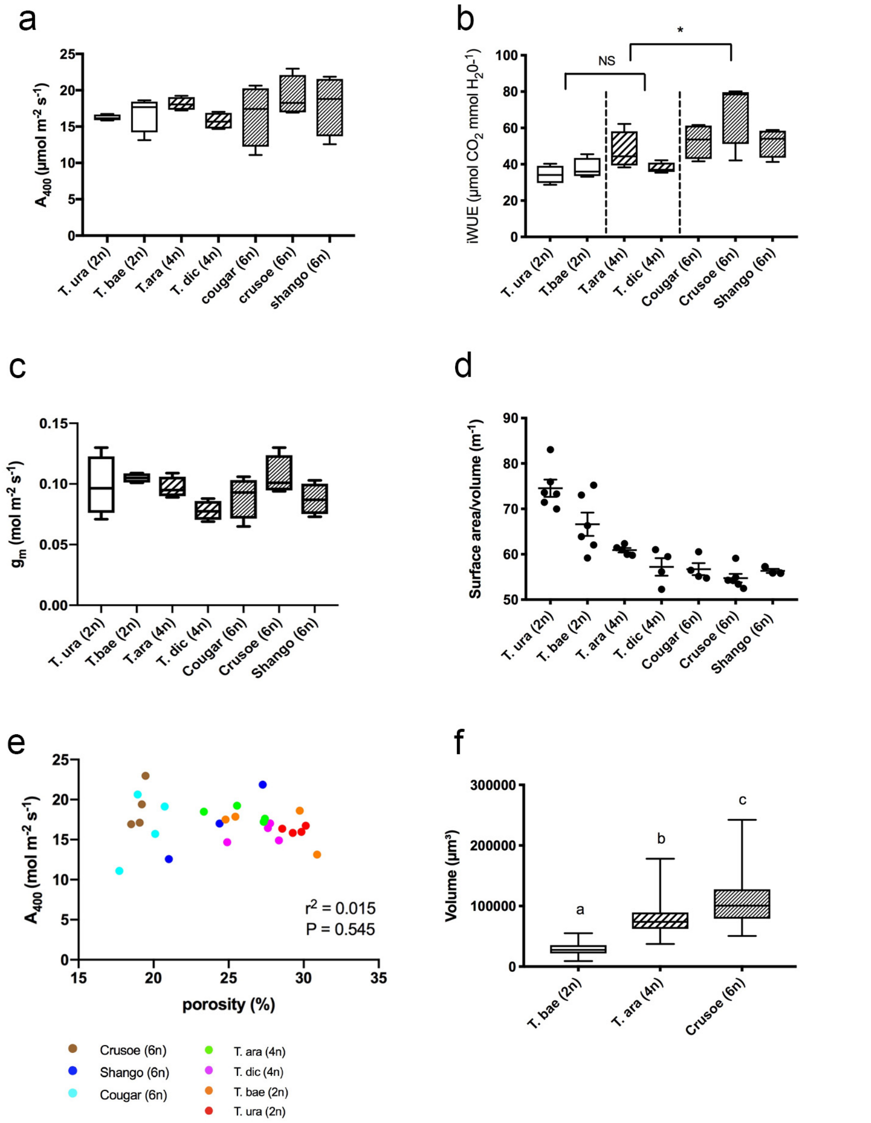

Supplementary Figure 5. Relationship of assimilation rate and water-use efficiency to wheat ploidy level

Assimilation rate (**a**) (ANOVA, $F_{(6,21)} = 0.779$, P = 0.596), iWUE **(b)** (ANOVA, $F_{(2,25)} = 10.76$, P = 0.0004), mesophyll conductance, g_m **(c)** (ANOVA, $F_{(6, 21)} = 1.958$ P = 0.118), exposed mesophyll surface area per volume (**d**) (ANOVA, $F_{(6, 27)} = 18.84$ P < 0.0001) are shown for all analyzed wheat lines. **(e)** For each analyzed wheat leaf, mean mesophyll porosity is plotted against mean assimilation A_{400} with ploidy level indicated for each point (see key). Results of correlation analysis are presented (Pearson r^2 value). (f) Cell volumes calculated from confocal microscopy stacks obtained from 2n, 4n, and 6n leaf samples (as indicated). ANOVA, $F_{(2, 173)} = 143.3$, P < 0.0001). For a-c, data are shown as box plots (25th-75th percentile, horizontal line = median) with whiskers indicating maximum and minimum values, n=6. For d, individual data points are shown, with mean value indicated with a horizontal bar. Results of a posthoc Tukey test comparing sequential ploidy levels are indicated in (b) with an asterisk when significant at the P < 0.05 level or NS when not significant. For (f), lines indicated with the same letter cannot be distinguished from each other at the 0.05 confidence limit (Tukey test).

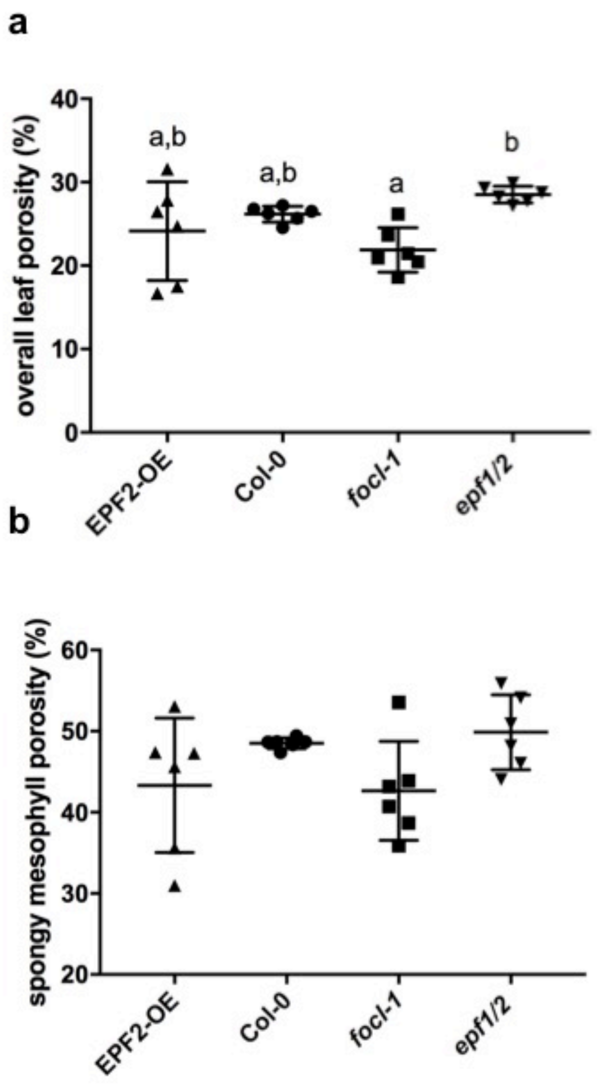

Supplementary Figure 6. Leaf porosity in Arabidopsis lines. Means and standard deviation of (**a**) overall leaf mesophyll and (**b**) spongy mesophyll porosity in Arabidopsis *EPF2-OE*, *Col-0*, *focl1-1* and *epf1epf2* lines, n = 5 for *Col-0,* n = 6 for mutants. ANOVA indicated a significant variance in mean porosity level in (a) ($F_{(3,20)}$ = 4.393, P = 0.016). Lines indicated with the same letter cannot be distinguished from each other at the 0.05 confidence limit (Tukey test). No significant variation in porosity of the spongy mesophyll layer was detected between lines ($F_{(3,20)}$ = 2.487, P = 0.090).

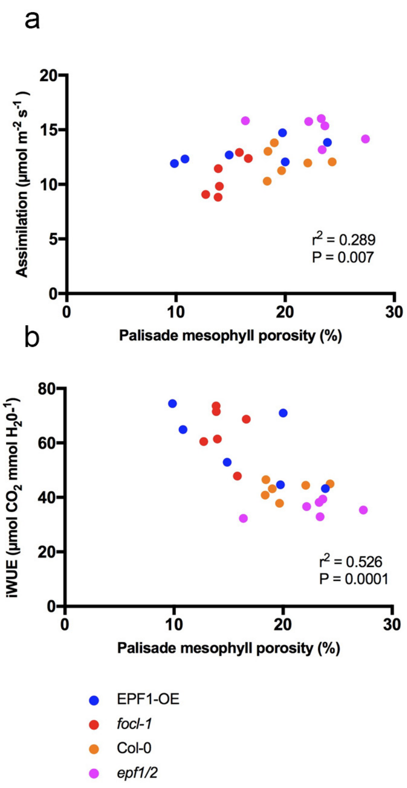

Supplementary Figure 7. Relationship of leaf porosity, assimilation rate and water-use efficiency in Arabidopsis lines. Palisade mesophyll porosity is plotted against **(a)** assimilation rate **(b)** iWUE for individual leaf samples from the four Arabidopsis lines, as indicated. Results of correlation analysis are presented (Pearson r^2 value).
