# Supplementary material for: Mesophyll porosity is modulated by the presence of functional stomata
Source: Nat Commun. 2019 Jun 27;10:2825. doi: 10.1038/s41467-019-10826-5 (PMC6597550; doi:10.1038/s41467-019-10826-5)
Supplement: Supplementary file 3 — Reporting Summary [file 41467_2019_10826_MOESM3_ESM.pdf]

nature research

Corresponding author(s): Andrew Fleming

Last updated by author(s): May 29, 2019

Reporting Summary

Nature Research wishes to improve the reproducibility of the work that we publish. This form provides structure for consistency and transparency in reporting. For further information on Nature Research policies, see Authors & Referees and the Editorial Policy Checklist.

Statistics

For all statistical analyses, confirm that the following items are present in the figure legend, table legend, main text, or Methods section.

n/a	Confirmed	
☐	☒	The exact sample size (*n*) for each experimental group/condition, given as a discrete number and unit of measurement
☐	☒	A statement on whether measurements were taken from distinct samples or whether the same sample was measured repeatedly
☐	☒	The statistical test(s) used AND whether they are one- or two-sided<br>*Only common tests should be described solely by name; describe more complex techniques in the Methods section.*
☐	☒	A description of all covariates tested
☒	☐	A description of any assumptions or corrections, such as tests of normality and adjustment for multiple comparisons
☐	☒	A full description of the statistical parameters including central tendency (e.g. means) or other basic estimates (e.g. regression coefficient) AND variation (e.g. standard deviation) or associated estimates of uncertainty (e.g. confidence intervals)
☐	☒	For null hypothesis testing, the test statistic (e.g. *F*, *t*, *r*) with confidence intervals, effect sizes, degrees of freedom and *P* value noted<br>*Give P values as exact values whenever suitable.*
☒	☐	For Bayesian analysis, information on the choice of priors and Markov chain Monte Carlo settings
☒	☐	For hierarchical and complex designs, identification of the appropriate level for tests and full reporting of outcomes
☐	☒	Estimates of effect sizes (e.g. Cohen's *d*, Pearson's *r*), indicating how they were calculated

Our web collection on statistics for biologists contains articles on many of the points above.

Software and code

Policy information about availability of computer code

Data collection	N/A
Data analysis	Prism 7 Graphpad for statistical analysis

For manuscripts utilizing custom algorithms or software that are central to the research but not yet described in published literature, software must be made available to editors/reviewers. We strongly encourage code deposition in a community repository (e.g. GitHub). See the Nature Research guidelines for submitting code & software for further information.

Data

Policy information about availability of data

All manuscripts must include a data availability statement. This statement should provide the following information, where applicable:

- Accession codes, unique identifiers, or web links for publicly available datasets
- A list of figures that have associated raw data
- A description of any restrictions on data availability

All datasets generated and/or analysed in this study are either provided in the Source Data file or are available on reasonable request from the corresponding author

Field-specific reporting

Please select the one below that is the best fit for your research. If you are not sure, read the appropriate sections before making your selection.

☒ Life sciences ☐ Behavioural & social sciences ☐ Ecological, evolutionary & environmental sciences

For a reference copy of the document with all sections, see nature.com/documents/nr-reporting-summary-flat.pdf

Life sciences study design

All studies must disclose on these points even when the disclosure is negative.

Sample size	Sample size for paired CT and gas exchange analysis was primarily determined by the logistics of growth space and the number of samples that can reasonably be processed by both techniques while maintaining high quality data collection (limiting data collection to a limited time of day and to comparable stages of leaf development, plus time taken to collect data/sample). The numbers used are comparable to the standards used by other researchers in the field.
Data exclusions	Data were only excluded where there was a clear technical problem with data collection, e.g., occasionally CT scans proved impossible to reliably analyse due to sample movements during the scanning process or during preparation for microscopy analysis samples were clearly physically damaged. These sample losses are reflected in the "n" values stated.
Replication	The paired experimental analysis of CT and gas exchange analysis, and the confocal and optical light microscopy analyses been successfully repeated within the limits sets by the restrictions on sample size described above.
Randomization	Plants for analysis were grown in a Conviron controlled environment chambers and rotated around the chamber to minimise position affects, as well as being located in the central portion of the chamber (again to minimize local light gradients that might influence results).
Blinding	The CT and physiology analyses were performed by different individuals on different days, as was a significant portion of the microscopy analysis, with samples of different treatments processed in a mixed fashion to obviate the risk both of user bias or shifts in the sensitivity of the analytical machinery on different days. Both CT and physiology data quality and analysis were, moreover, checked/repeated by independent researchers, with analyses traceable back to original time/date of sample collection linked to individual codes for each sample.

Reporting for specific materials, systems and methods

We require information from authors about some types of materials, experimental systems and methods used in many studies. Here, indicate whether each material, system or method listed is relevant to your study. If you are not sure if a list item applies to your research, read the appropriate section before selecting a response.

Materials & experimental systems

n/a	Involved in the study	
☒	☐	Antibodies
☒	☐	Eukaryotic cell lines
☒	☐	Palaeontology
☒	☐	Animals and other organisms
☒	☐	Human research participants
☒	☐	Clinical data

Methods

n/a	Involved in the study	
☒	☐	ChIP-seq
☒	☐	Flow cytometry
☒	☐	MRI-based neuroimaging
